# MRI findings for differentiating benign and malignant soft tissue tumors: a narrative review- Part 1: diagnostic performance

**DOI:** 10.1007/s00256-026-05223-1

**Published:** 2026-05-01

**Authors:** Mahad Rehman, Shyam Ramachandran, Muaz Wahid, Aayush Sharma, Majid Chalian, Gitanjali Bajaj, Jim S Wu, Hillary Garner, Jonathan Samet, Shivani Ahlawat, Kambiz Motamedi, Ty Subhawong, Mark Murphey, Avneesh Chhabra

**Affiliations:** 1 UT Southwestern Medical Center, TX Dallas, USA; 2 Texas A&M School of Medicine, College Station, Bryan, TX USA; 3 Rosalind Franklin University of Medicine and Science, North Chicago, IL USA; 4 Department of Radiology, University of Washington, Seattle, WA USA; 5 Department of Radiology, University of Arkansas for Medical Sciences, Little Rock, AR USA; 6 Department of Radiology, Brigham and Women’s Hospital, Harvard Medical School, Boston, MA USA; 7 Department of Radiology, Mayo Clinic, Jacksonville, FL USA; 8 Department of Radiology, Northwestern University Feinberg School of Medicine, Chicago, IL USA; 9 Russell H. Morgan Department of Radiology and Radiological Science, Johns Hopkins University, Baltimore, MD USA; 10 Department of Radiology, University of California, Los Angeles, CA USA; 11 Department of Radiology, University of Miami Miller School of Medicine, Miami, FL USA; 12 Uniformed Services University of the Health Sciences, Bethesda, MD USA; 13 ACR (American College of Radiology Institute for Radiologic Pathology, AIRP) Silver Spring, Silver Spring, MD USA; 14 Walter Reed National Military Medical Center, Bethesda, MD USA; 15 UTSW, Dallas, TX USA; 16 Johns Hopkins University, Baltimore, USA MD; 17 Walton Center of Neurosciences, Liverpool, UK; 18 Chicago Medical School, Rosalind Franklin University, North Chicago, USA

**Keywords:** Soft tissue sarcoma, MRI, Diffusion weighted imaging, DWI, Diagnostic performance

## Abstract

This review aims to assess the diagnostic performance of MRI in distinguishing benign from malignant soft tissue tumors and evaluate conventional MRI sequences (Aim 1), as well as assess diffusion-weighted imaging (DWI) for tumor differentiation (Aim 2A) and tumor grading (Aim 2B). PubMed, Scopus, Embase, and CENTRAL were searched from inception to March 2025 using keywords like “MRI,” “soft tissue sarcoma,” “sensitivity,” “specificity,” and “diffusion.” Studies with ≥ 20 cases reporting sensitivity and specificity for MRI or DWI were included for Aims 1/2A; studies on DWI for grading were included for Aim 2B. Four reviewers screened titles, abstracts, and full texts, resolving disagreements by consensus. A total of 224 studies were initially identified, and 34 were included for final analysis (Aim 1: 16, Aim 2A: 12, Aim 2B: 6). Conventional MRI (Aim 1) demonstrated sensitivities of 61–100%, specificities of 37–98%, and high negative predictive values (up to 98%) but lower positive predictive values (38–60%). DWI (Aim 2A) showed sensitivities of 73–100%, specificities of 63–94%, and lower ADC values in malignant tumors. ADC values were typically lower in high-grade tumors (Aim 2B). Overall, MRI appears to offer high diagnostic performance in excluding malignancy but lower positive predictive value and thus should be paired with histopathology or supplemental imaging. Integrating DWI into MRI protocols may enhance diagnostic accuracy by increasing specificity and confidence in distinguishing between benign and malignant tumors. ADC values from diffusion-weighted imaging were inversely correlated with tumor grade, suggesting utility in differentiating between low- and high-grade sarcomas.

## Introduction

Soft tissue sarcomas (STS) represent a complex and rare subset of cancers, arising from mesenchymal cells across various body sites. Accounting for approximately 1% of all adult tumors, STS present challenges in detection and diagnostic characterization from other similar-appearing benign soft tissue tumors due to their heterogeneity and rarity [1, 2]. Over 70 distinct sarcoma subtypes have been identified in the more recent World Health Organization (WHO) classification of soft tissue tumors, contributing to diverse clinical presentations and prognoses, but often complicating diagnostic and therapeutic pathways [3].

The etiology of most STS remains elusive, with only a fraction being attributable to identifiable risk factors, such as prior radiation exposure or underlying genetic derangement. They can manifest anywhere in the body, with a predilection for the extremities, trunk, and retroperitoneum [1]. The age distribution of STS incidence exhibits a bimodal pattern, with a notable peak in adults around 60 years of age and a smaller peak in early childhood, between 0 and 5 years [4].

Treatment strategies for STS typically involve surgical resection, which can be curative when tumors are localized [3, 5]. The efficacy of neoadjuvant therapies, including chemotherapy and radiotherapy, however, varies and is often limited. Having negative surgical margins is critical for disease-free survival, with limb salvage being a priority in extremity cases to improve functional outcomes [5]. For patients with tumors involving major vessels, surgical decisions between wide en-bloc excision and vessel-sparing techniques are informed by advanced imaging studies, including MRI and CT angiography [5]. Pre-operative imaging can play an important role in surgical planning by informing surgeons of adjacent anatomy with particular emphasis on avoiding neurovascular structures, especially using contrast-perfusion and delayed contrast imaging. Ultimately, this approach can deliver better outcomes for patients undergoing resection of tumors.

The prognosis of STS is predominantly influenced by the histological grade of malignancy, with grading systems like the FNCLCC (Federation Nationale des Centres de Lutte Contre le Cancer) and NCI (National Cancer Institute) systems aiding in prognostication and treatment planning [6]. While the gold standard for determining the grade of malignancy is core needle or surgical biopsy, this carries certain risks, including potential tumor cell spread and misestimation of malignancy due to intratumoral heterogeneity [3]. Due to the complexity and rarity of STS, the rate of diagnostic inaccuracy ranges between 20 and 30% [7–9].

Imaging plays a pivotal role in the detection and management of benign and malignant soft tissue lesions. Magnetic resonance imaging (MRI) is the preferred modality for providing detailed information crucial for localization, characterization, and defining the stage of the disease for extremity soft tissue lesions [10]. Research has demonstrated that MRI features, including the presence of necrosis, edema around the tumor, and variability in signal on both T1- and T2-weighted images, as well as the lesion’s depth and its relationship to fascia, are significant indicators that help distinguish benign from malignant soft tissue tumors [11, 12]. Therefore, sarcoma can often be excluded on MRI when benign features are present, decreasing the risk of unnecessary workup. Accurate diagnosis of sarcoma, however, remains challenging due to overlapping imaging appearances between sarcomas and certain benign tumors on cross-sectional imaging.

This narrative review is aimed at evaluating and consolidating the limited literature that exists regarding the diagnostic accuracy of MRI for determining benign versus malignant soft tissue tumors. Our first aim focuses on analyzing the reported sensitivity and specificity of conventional MRI sequences in identifying benign versus malignant tumors. Aim 2 focuses on the role of diffusion weighted MR imaging (DWI) and is further broken down into two sub-aims, with the goal of analyzing the reported sensitivity and specificity of DWI in identifying benign versus malignant tumors (Aim 2 A) and its role in tumor grading (Aim 2B). Most of the current literature on the imaging of STS focuses on tumor findings and description in different case series. By consolidating diagnostic accuracy findings across studies, we hope to gain a better understanding of the use of MRI in characterizing soft tissue lesions. This type of work can serve as a reference for future iterations and a go-to composition when new data becomes available on the diagnostic performance of MRI or advanced imaging, such as DWI characteristics of a soft tissue tumor.

## Literature search strategy

### Review protocol

This narrative review was conducted using a structured literature search approach guided by PRISMA principles.

### Search strategy

Given the narrative nature of this review, an Institutional Review Board (IRB) approval was not applicable. The radiologist authors as part of the American College of Radiology committee of experts on Soft-tissue RADS project were engaged in this project with 4 medical students to perform an extensive search of the existent literature. PubMed, Scopus, Embase, and The Cochrane Central Register of Controlled Trials (CENTRAL) were searched for relevant studies using selected keywords. These chosen keywords included a combination of “MRI,” “magnetic resonance imaging,” “sarcoma,” “soft tissue sarcoma,” “accuracy,” “sensitivity, “specificity,” “grade,” “grading,” and “diffusion.” We also substituted “soft tissue sarcoma” and “sarcoma” with all applicable subtypes of STS as outlined by the most recent 2020 WHO histological typing and applied multiple combinations of these keywords with appropriate Boolean operators (OR/AND) to each of the four databases. The search involved articles from 1985 for all databases through March 2025 with no language limitations. Reference lists of included studies were also reviewed for additional relevant studies.

### Study eligibility

For aim 1 and 2 A, we included all studies that had 20 or more cases analyzing the sensitivity and specificity of MRI for differentiating between benign and malignant soft tissue tumors. For aim 2B, we included all scientific studies that analyzed the role of diffusion grading for STS. For all aims, we searched for studies performed on the general category of soft tissue tumors as well as all STS subtypes as classified by the WHO histological typing [3]. This included randomized controlled trials, prospective cohort studies, retrospective cohort studies, and case–control studies. Case reports, animal studies, cadaveric studies, and author replies were excluded.

### Study selection

Four independent medical student reviewers conducted an initial assessment of the titles and abstracts from the search results. Studies unrelated to the research subject were excluded. The remaining studies deemed potentially relevant underwent a comprehensive full-text analysis to ascertain their suitability based on the inclusion criteria. All articles were evaluated by expert musculoskeletal radiologists as part of the American College of Radiology Soft-Tissue RADS committee. Disagreements were resolved by consensus among the authors. All articles are archived in portable document format within the ACR repository.

### Data extraction

Data were extracted pertaining to each individual aim for which the study was included. For all aims, sensitivity and specificity were noted in our review. We also collected additional information about each study’s characteristics. Further information was extracted for Aim 2B relating to ADC values. Data from each aim were tabulated for easier viewing in our review.

## Findings

### Included studies

**Fig. 1:**
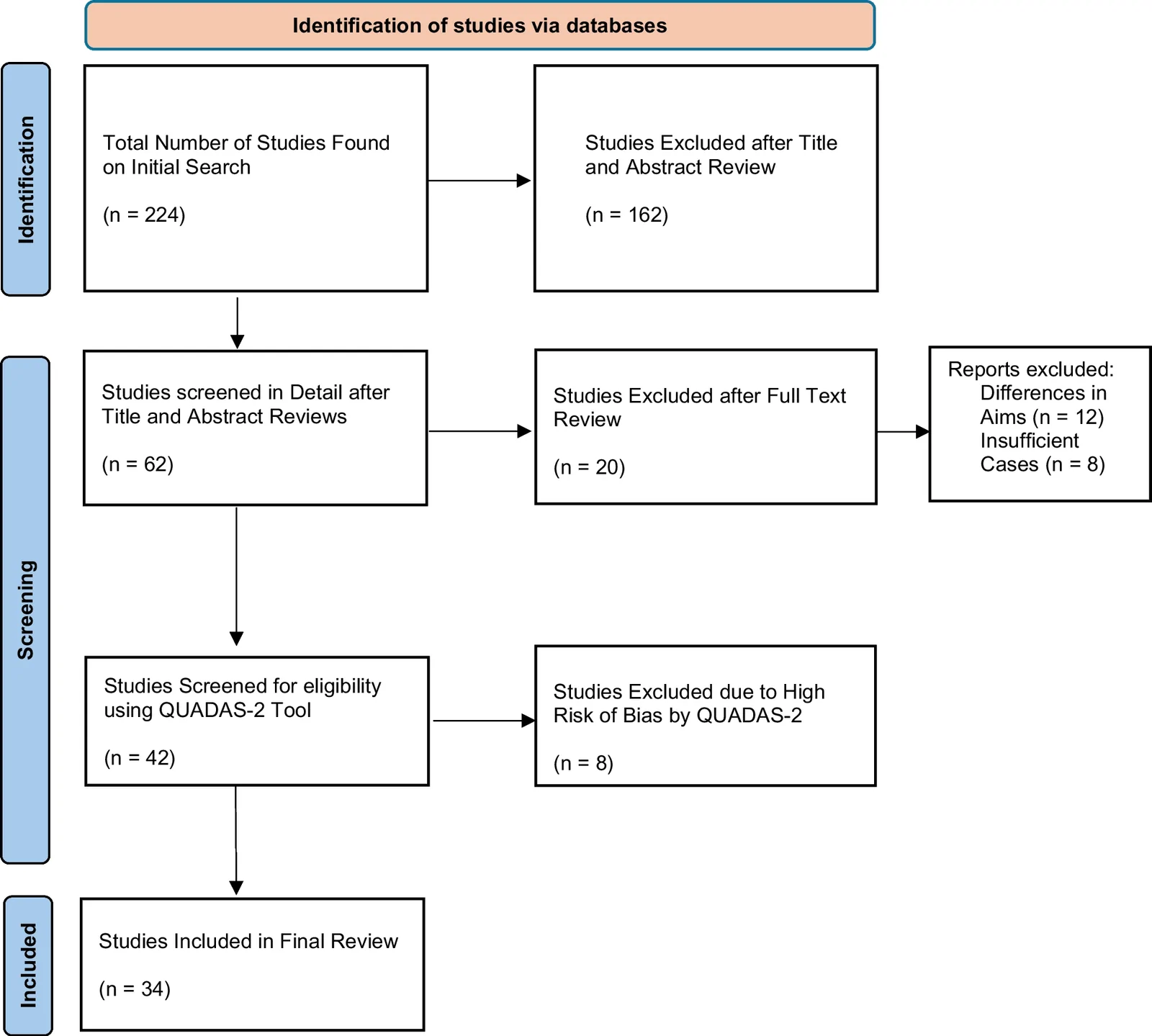
Flowchart of literature review process

A total of 224 studies were identified by our initial search. Sixty-two relevant articles were assessed for eligibility. Of these, 12 studies were out of the scope of our aims and 8 did not have enough included cases, with 42 studies remaining. After excluding for bias, 34 studies were included in our final review, 16 studies were included for aim 1, 12 studies for aim 2A, and 6 studies for aim 2B. Utilizing the PRISMA 2020 guidelines, the process for study inclusion can be seen in Fig. 1. [13] Table 1 includes a summary of study characteristics such as country performed, single vs multi-institution, number of tumors, tumor type, and Oxford Level of Evidence. The included studies were from a wide range of countries with adequate sample sizes.

**Table 1:** Study characteristics

	Study	Year	Country	Single vs multi-institutional	Tumor type	Number of tumors	Imaging category	Oxford level of evidence
Aim 1	Gielen et al. [12]	2004	Belgium	Multi	Benign vs malignant	548 total (425 benign, 123 malignant)	MRI	II
	Berquist et al. [14]	1990	USA	Single	Benign vs malignant	95 total (50 benign, 45 malignant)	MRI	III
	Moulton et al. [15]	1995	USA	Single	Benign vs malignant	225 total (179 benign, 46 malignant)	MRI	III
	De Schepper et al. [16]	1992	Belgium	Single	Benign vs malignant	141 total (84 benign, 57 malignant)	MRI	III
	Sen et al. [17]	2010	India	Single	Benign vs malignant	55 total (32 benign, 23 malignant)	MRI	II
	Shu et al.[18]	2022	China	Single	Benign vs malignant	120 total (49 benign, 71 malignant)	MRI + DWI	III
	Chung et al. [19]	2012	South Korea	Single	Benign vs malignant	266 total (164 benign, 102 malignant; 59 adipocytic)	MRI	III
	Chhabra et al. (ST-RADS) [20]	2022	USA	Multi	Benign vs malignant	200 total (100 adipocytic, 50 T2-hyperintense, 50 T2-hypointense)	MRI	II
	Coran et al. [21]	2017	Italy	Single	Lipomatous tumors	54 total	MRI	III
	Brisson et al. [22]	2013	UK	Single	Lipomatous soft-tissue tumors	87 total (54 lipoma, 33 ALT/WDL)	MRI	III
	Doyle et al. [23]	2008	New Zealand	Single	Lipoma vs ALT/WDLS	51	MRI	III
	Galant et al. [24]	2003	Spain	Single	Lipoma vs liposarcoma	92 total (60 benign, 32 WDL	MRI	III
	Cairncross et al. [25]	2019	UK	Single	Lipoma vs ALT/WDLS	248 total (167 lipoma, 81 ALT)	MRI	III
	Gaskin et al. [26]	2004	USA	Single	Lipoma vs WDLS	126 total (108 lipoma, 18 WDL)	MRI	III
	Wasa et al. [27]	2010	Japan	Single	PNST	61 total (20 benign, 41 malignant)	MRI	III
	Wilson et al. [28]	2021	Multiple	Multi	PNST	798 total (546 benign, 252 malignant)	MRI ± DWI	I
Aim 2A	Hassanien et al. [29]	2018	Egypt	Single	Benign vs malignant	45 total (24 benign, 21 malignant	DWI	II
	Einarsdóttir et al.[30]	2004	Sweden	Single	Benign vs malignant	29 total (16 benign, 13 sarcomas)	DWI	II
	van Rijswijk et al.[31]	2002	Netherlands	Single	Benign vs malignant	22 total (12 benign, 10 malignant)	DWI	II
	Wang et al.[32]	2021	Multiple	Multi	Benign vs malignant	1319 (696 benign, 623 malignant)	DWI	I
	Nagata et al. [33]	2008	Japan	Single	Benign vs malignant	88 total (44 benign, 36 malignant, 8 intermediate)	DWI	III
	Nassef et al. [34]	2021	Egypt	Single	Benign vs malignant	20 total (11 benign, 9 malignant)	DWI	II
	Hong et al.[35]	2019	South Korea	Single	Benign vs malignant	45 total	MRI + DWI	III
	Lee et al.[36]	2020	South Korea	Single	Benign vs malignant	67 tumors total (35 benign, 32 malignant)	MRI + DWI	III
	Zampa et al.[37]	2023	Italy	Single	Benign vs malignant	37 total (16 malignant, 21 benign)	DWI	III
	Demehri et al. [38]	2014	USA	Single	Benign and malignant PNSTs	31 total (9 malignant, 22 benign)	MRI + DWI	II
	Nakajo et al. [39]	2018	Japan	Single	Benign PNST and soft tissue sarcomas (STS)	56 total (25 benign, 31 malignant)	DWI	III
	Li et al.[11]	2023	China	Single	Benign vs malignant	71 malignant tumors	MRI + DWI	III
Aim 2B	Assy et al.[40]	2022	Egypt	Single	Benign vs malignant	52 total (24 low grade, 28 high grade)	MRI + DWI	III
	Chhabra et al. [41]	2019	USA	Single	Malignant tumors	51	MRI + DWI	III
	Manikis et al. [42]	2021	Greece	Single	Soft tissue sarcoma	60	DWI	III
	Lee et al. [36]	2019	South Korea	Single	Malignant soft tissue tumors	36	MRI + DWI	III
	Hong et al.[35]	2020	South Korea	Single	Malignant soft tissue tumors	42	MRI + DWI	III
	Gowda et al. [43]	2023	USA	Single	Malignant soft tissue tumors	78	DWI	III

- Aim 1
  - Diagnostic accuracy of MRI in STS

**Table 2:** Major findings from included studies for Aim 1: diagnostic performance of MRI for benign versus malignant soft tissue tumors

Study (first author et al., year)	Overall sensitivity (%)	Overall specificity (%)	Imaging modalities	Number of tumors (***N***)	Tumor type	Number of readers	Inter-reader analysis	Intra-reader analysis	Study summary
Gielen et al., 2004 [12]	93	82	MRI	548 total (425 benign, 123 malignant)	Benign vs malignant	2	NA	NA	Sensitivity of 93%, specificity of 82%, NPV of 98%, PPV of 60%, and accuracy of 85% in distinguishing malignant from benign lesions
Berquist et al., 1990 [14]	94	90	MRI	95 total (50 benign, 45 malignant)	Benign vs malignant	3	NA	NA	Sensitivity of 94%, specificity of 90%, NPV of 94%, and PPV of 87% for detecting malignant lesions with an overall accuracy of 90%. For benign lesions, the values were as follows: sensitivity 88%, specificity 90%, NPV 87%, PPV 94%, and accuracy 90%.
Moulton et al., 1995 [15]	78	89	MRI	225 total (179 benign, 46 malignant)	Benign vs malignant	5	NA	NA	Sensitivity of 78%, specificity of 89%, NPV of 94%, and PPV is 65% for diagnosing malignant lesions. Higher prevalence of benign lesions influenced specificity and NPV.
De Schepper et al., 1992 [16]	81	81	MRI	141 total (84 benign, 57 malignant)	Benign vs malignant	NA	NA	NA	Highest sensitivity for predicting malignancy: “absence of low signal intensity on T2” (100%), “mean diameter > 33 mm” (90%), and “inhomogeneous signal on T1” (88%). Highest specificity: “evidence of necrosis” (98%), “bone or neurovascular involvement” (94%).
Sen et al., 2010 [17]	83	81	MRI	55 total (32 benign, 23 malignant)	Benign vs malignant	NA	NA	NA	Sensitivity of 83%, specificity of 81%, PPV of 76%, NPV of 87%, and overall accuracy was 82% for diagnosing malignant lesions
Shu et al., 2022 [18]	92% (US), 87% (MRI), 91% (combined)	72% (US), 76% (MRI), 82% (combined)	US, MRI	120 total (49 benign, 71 malignant)	Benign vs malignant	2	NA	NA	MRI demonstrated a sensitivity of 87% and specificity of 76% for diagnosing malignant lesions. Combining US and MRI improved diagnostic accuracy with sensitivity of 91%, specificity of 82%, and overall accuracy of 93%.
Chung et al., 2012 [19]	64	85	MRI	266 total (164 benign, 102 malignant; 59 adipocytic)	Benign vs malignant	2	NA	NA	Evaluated depth, size, and T2W signal heterogeneity of biopsied/excised tumors. Sensitivity was 64%, specificity was 85%, PPV was 32%, NPV was 59%, and overall accuracy was 77%. Tumor depth not independent predictor (adjusted OR: 1.11 (0.58–2.13)
Chhabra et al., 2022 [20]	64–96	63–71	MRI, DWI	200 total (100 adipocytic, 50 T2-hyperintense, 50 T2-hypointense)	Benign vs malignant	8	ICC: 0.72 (adipocytic), 0.69 (T2-hyperintense), 0.51 (T2-hypointense)	NA	ST-RADS classification system demonstrated different sensitivity and specificity, based on tumor types. For adipocytic tumors: sensitivity 96%, specificity 63%. T2-hyperintense tumors: sensitivity 93%, specificity 71%. T2-hypointense tumors: sensitivity 64%, specificity 84%. Overall AUC = 0.79–0.89
Coran et al., 2017 [21]	100	75	MRI	54 total	Lipomatous tumors	2	100% (kappa = 0.99)	NA	Sensitivity of 100%, specificity of 75%, PPV of 96%, and NPV of 100% for detecting liposarcomas. Significant MRI findings for malignancy were septa > 2 mm thick (*p* < 0.05), and signal inhomogeneity (*p* < 0.05)
Brisson et al., 2013 [22]	90.9	37	MRI	87 total (54 lipoma, 33 ALT/WDL)	Lipomatous soft-tissue tumors	2	Kappa = 0.71	Kappa = 0.89	MRI differentiated ALT/WDL with a sensitivity of 90.9%, specificity of 37.0%, PPV of 46.9%, and NPV of 86.9%. Reliable discriminators of ALT/WDL included overall lesion size (size of lesion > 10 cm: *p* = 0.011), lipomatous content (*p* = 0.002), and solid nodules > 1 cm, though MRI appearances overlapped
Galant et al., 2003 [24]	Sensitivity of fs T2/STIR: 100%; sensitivity for T1W: 96.9%	Specificity for fs T2/STIR: 88.3%; specificity for T1W: 76.7%	MRI	92 total (60 benign, 32 WDL)	Lipoma vs liposarcoma	2	NA	NA	Using fluid-sensitive (fsT2W or STIR) images: sensitivity 100%, specificity 88.3% for detecting WDLS. PPV was 82.1%, NPV was 100%. The values were higher than T1W images
Doyle et al., 2008 [23]	94–100	64–76	MRI	51 total	Lipoma vs ALT/WDLS	2	Kappa: 0.87 (T1W), 0.88 (fluid-sensitive), 0.84 (combined sequences)​	NA	Sensitivity for detecting ALT/WDL: on T1W sequence = 100% and 94% for the two observers. On fluid-sensitive sequences: 100% sensitivity for both Specificity: on T1W sequence = 76% and 64% and on fluid-sensitive sequence = 70% and 73%. Combining sequences: sensitivity 100%, specificity 70% and 61%
Gaskin et al., 2004 [26]	100	83	MRI	126 total (108 lipoma, 18 WDL)	Lipoma vs WDLS	7	NA	NA	For WDLS, sensitivity of 100%, specificity of 83%, PPV of 38%, NPV of 100%, and overall accuracy of 84%
Cairncross et al., 2019 [25]	90.1	51.5	MRI	248 total (167 lipoma, 81 ALT)	Lipoma vs ALT/WDLS	NA	NA	NA	For predicting ALT, sensitivity of 90.1%, specificity of 51.5%, PPV of 47.4%, NPV of 91.5%, and overall accuracy of 64.1%. No ALT < 5 cm
Wasa et al., 2009 [27]	61	90	MRI	61 total (20 benign, 41 malignant)	PNST	2	NA	NA	Important MRI characteristics for distinguishing MPNST from neurofibroma included larger tumor size (median 9.9 cm vs. 5 cm, *p* = 0.029), peripheral enhancement pattern (*p* = 0.006), presence of a perilesional edema-like zone (*p* = 0.005), and intratumoral cystic lesions (*p* = 0.02). The presence of two or more of these features increased diagnostic accuracy for diagnosing MPNST (sensitivity of 61% and specificity of 90%)
Wilson et al. 2021 [28]	68% (52–80%) for combined features; 88% (74–95%) for diffusion restriction​	93% (85–97%) for combined features; 94% (89–96%) for diffusion restriction	MRI and DWI	798 total (546 benign, 252 malignant)	PNST	2	Kappa = 0.64–0.94	NA	Pooled sensitivity for combined MRI features was 88% (95% CI, 74–95%) and specificity was 94% (95% CI, 89–96%). Diffusion restriction showed high sensitivity (100%, 95% CI, 28–100%). Combining size with diffusion restriction improved sensitivity to 100%

*ALT* atypical lipomatous tumor, *AUC* area under the curve, *CI* confidence interval, *DWI* diffusion-weighted imaging, *ICC* intraclass coefficient, *MM* millimeter, *MPNST* malignant peripheral nerve sheath tumor, *PNST* peripheral nerve sheath tumor, *PPV* positive predictive value, *NA* not available, *NPV* negative predictive value, *WDLS* well-differentiated liposarcoma

The evaluation of MRI’s diagnostic accuracy across various STS revealed significant variability in sensitivity and specificity when distinguishing between benign and malignant lesions. All results are summarized in Table 2. Across 16 studies included for Aim 1, sensitivity ranged from 61 to 100%, specificity ranged from 37 to 97%, PPV ranged from 32 to 94%, and NPV ranged from 87 to 100%. When examining specific MRI parameters, signal intensity, size, and depth differed between benign and malignant masses, and depth was not an independent predictor. The combined use of these parameters yielded a sensitivity of 64%, specificity of 85%, and overall accuracy of 77% [19]. Alongside MRI findings, Chhabra et al. introduced the ST-RADS classification system to stratify musculoskeletal tumors [20]. The study found that the sensitivity and specificity of MRI varied by tumor type, with adipocytic tumors showing a sensitivity of 96% and specificity of 63%, T2-hyperintense tumors with a sensitivity of 93% and specificity of 71%, and T2-hypointense tumors showing a sensitivity of 64% and specificity of 84%. The area under the curve (AUC) ranged from 0.79 to 0.89, indicating good diagnostic accuracy across different tumor types [20]. Lipomatous tumors had variable diagnostic accuracy for MRI. Sensitivity ranged from 90 to 100%, specificity ranged from 37 to 83%. Two studies included on peripheral nerve sheath tumors demonstrated a sensitivity range of 61–100% and specificity range of 90–94%.

- Aim 2A
  - of DWI for STS

**Table 3:** Major findings of included studies for aim 2A: diagnostic performance of DWI with apparent diffusion coefficient (ADC) for benign versus malignant soft tissue tumors

Study (first author et al., year)	Imaging modalities, sequence	Sensitivity (%)	Specificity (%)	Number of tumors (N)	Tumor type	Number of readers	Inter-reader analysis	Intra-reader analysis	Minimum ADC (× 10^−3^ mm^2^/s)	Mean/median ADC (×10^−3^ mm^2^/s)	Maximum ADC (× 10^−3^ mm^2^/s)	Summary of findings
Hassanien et al., 2018 [29]	MRI, DWI	73	91.7	45 total (24 benign, 21 malignant	Benign vs malignant	NA	NA	NA	0.3	1.53 (benign), 0.84 (malignant)	2.7	Overall, benign tumors had ADC values above 1.28 and malignant tumors had ADC values below 1.1 (*p* < 0.05)
Einarsdóttir et al., 2004 [30]	MRI, DWI	NA	NA	29 total (16 benign, 13 sarcomas)	Benign vs malignant	NA	NA	NA	0.9	1.8 (benign), 1.7 (malignant)	2.3	Mean ADC values of benign lesions and sarcomas overlapped significantly, making differentiation challenging based on ADC values alone
van Rijswijk et al., 2002 [31]	MRI, DWI	NA	NA	22 total (12 benign, 10 malignant)	Benign vs malignant	NA	NA	NA	0.19	1.71 (benign), 1.08 (malignant)	2.9	True diffusion coefficients of malignant tumors were significantly lower than those of benign masses. (*p* < 0.05)
Wang et al., 2021 [32]	MRI, DWI, IVIM, DKI	80	63	1319 (696 benign, 623 malignant)	Benign vs malignant	NA	NA	NA	0.76	Benign: 1.92, malignant: 0.94	2.9	Malignant tumors had significantly lower mean ADC values (0.94 × 10⁻^3^ mm^2^/s) compared to benign tumors (1.92 × 10⁻^3^ mm^2^/s, *p* < 0.05). IVIM parameters, especially the diffusion coefficient (D), showed superior performance with an AUC of 0.874
Nagata et al., 2008 [33]	MRI, DWI	NA	NA	88 total (44 benign, 36 malignant, 8 intermediate)	Benign vs malignant	NA	NA	NA	0.4	2.08 (myxoid), 1.31 (benign nonmyxoid), 0.94 (malignant nonmyxoid)	2.28	The ADC value for nonmyxoid tumors was significantly lower in malignant tumors (*p* < 0.001). ADC values > 1.35 × 10^−3^ mm^2^/s were suggestive of benign tumors
Nassef et al., 2021[34]	MRI, DWI	100	81.8	20 total (11 benign, 9 malignant)	Benign vs malignant	NA	NA	NA	0.54 (sarcoma)	1.58 (benign), 0.70 (malignant)	2.44 (myxoma)	Malignant tumors had significantly lower ADC values than benign tumors (*p* < 0.001). A cut-off ADC value of 1.14 × 10^−3^ mm^2^/s had an AUC of 0.91
Hong et al., 2019 [44]	MRI, DWI	100% (reader 1), 97% (reader 2)	67% (reader 1), 42% (reader 2)	45 total	Benign vs malignant	2	Kappa = 0.646 (conventional MRI) Kappa = 0.496 (conventional MRI + DWI)	Kappa = 0.871 for MRI + DWI	NA	NA	NA	Addition of DWI to conventional MRI significantly improved specificity (*p* = 0.008 for reader 1). AUC for MRI + DWI was significantly higher: 0.890 vs 0.678 (*p* = 0.0123) for reader 1 and 0.846 vs 0.640 (*p* = 0.0305) for Reader 2
Lee et al., 2020​ [36]	Multiparametric MRI-IVIM-DWI and DCE-MRI	87	73	67 tumors total (35 benign, 32 malignant)	Benign vs malignant	2	ICC = 0.95–0.98	NA	NA	1.17 (malignant)	1.47 (benign)	ADC values were significantly lower in malignant tumors (*p* < 0.05)
Nakajo et al., 2016 [39]	MRI, DWI	85.7%	92%	56 total (25 benign, 31 malignant)	Benign PNST and soft tissue sarcomas (STS)	2	ICC = 0.906–0.997	NA	1.09	1.88 (benign PNST), 1.44 (STS)​	2.27 (BPNST), 1.86 (STS)	The 25th percentile ADC showed the highest AUC (0.773) for differentiating Benign PNST from STS (*p* < 0.001). CV and skewness were significantly higher in STS compared to benign PNST (*p* < 0.05)
Demehri et al., 2014 [38]	MRI, DWI, DCE-MRI	100	77	31 total (9 malignant, 22 benign)	Benign and malignant PNSTs	2	(ICC = 0.82–0.89)	NA	0.47 (malignant)	Benign: 1.08 ± 0.26 × 10^−3^ mm^2^/s, malignant: 0.47 ± 0.32 × 10^−3^ mm^2^/s	NA	Tumor diameter and minimum ADC significantly differentiated benign from malignant PNSTs (*p* < 0.001). Average tumor diameter ≥ 4.2 cm and minimum ADC ≤ 1.0 × 10^−3^ mm^2^/s had 100% sensitivity for malignancy
Zampa et al., 2022 [37]	MRI, DWI	100	80	37 total (16 malignant, 21 benign)	Benign vs malignant	2	Kappa = 0.755	NA	0.5	1.31 (lesion), 1.70 (scar)	2.6	DWI had higher diagnostic confidence than conventional MRI (*p* < 0.001). ADC values for recurrent lesions were significantly lower than for scar tissue (*p* = 0.000)
Li et al., 2022 [11]	MRI, DWI, DCE	NA	NA	71 malignant tumors	Benign vs malignant	2	ICC = 0.826–0.966	NA	NA	Mean: 1.58, median: 1.44	1.827	Using histogram metrics from DWI and DCE-MRI parameters with HIF-1alpha expression in STS. Higher T2W image heterogeneity and more than 50% necrotic area were associated with higher HIF-1alpha expression. Significant (*p* < 0.001) correlations were found between ADC skewness, Ktrans, and HIF-1alpha expression

*ADC* apparent diffusion coefficient, *AUC* area under the curve, *CI* confidence interval, *CM* centimeter, *DCE-MRI* dynamic contrast enhanced MRI, *DKI* diffusion kurtosis imaging, *DWI* diffusion-weighted imaging, *ICC* intraclass coefficient, *IVIM* intravoxel incoherent motion, MM millimeter, *MPNST* malignant peripheral nerve sheath tumor, *PNST* peripheral nerve sheath tumor, *PPV* positive predictive value, *NA* not available, *NPV* negative predictive value, *STS* soft tissue sarcoma, *CV* coefficient of variation

Table 3 shows the summary of major findings for the diagnostic performance of DWI with apparent diffusion coefficient (ADC) for benign versus malignant soft tissue tumors. Across the 12 studies included for Aim 2 A, sensitivity ranged from 73 to 100% and specificity ranged from 73 to 92%. A review of these studies demonstrated variability in the utility of ADC for differentiation. Some studies found that malignant soft tissue tumors demonstrated significantly lower ADC values compared to benign tumors [34]. Other studies demonstrated overlap in values between both [30]. When examining perfusion-corrected DWI, malignant tumors demonstrated lower true diffusion coefficients [31]. Intravoxel incoherent motion (IVIM) and diffusion kurtosis imaging (DKI) were also studied, demonstrating higher accuracy in non-myxoid tumors. Overall, DWI using ADC values achieved a pooled sensitivity of 0.80 and specificity of 0.63 for differentiating between benign and malignant soft tissue tumors [32]. DWI may also have a benefit for surgical planning by improving identification of margins and post-operative monitoring [35, 37]. When looking at peripheral nerve sheath tumors, malignant forms had significantly lower ADC values compared to benign tumors [38]. ADC histogram analysis can effectively differentiate between benign peripheral neurogenic tumors and STS with malignant PNSTs having lower ADC values and higher skewness and coefficient of variation, reflecting the greater heterogeneity and cellularity of malignant tumors [39].

- Aim 2B
  - Discriminatory power of ADC measurements

Table 4 shows the summary of major findings for the use of DWI in grading of STS. Overall, numerous studies demonstrated that ADC values can help differentiate tumor grade, with higher-grade tumors demonstrating lower ADC values. The lower ADC values in higher-grade tumors indicate increased cellularity, which is characteristic of more aggressive malignancies. Variations in cellular composition may also assist in differentiating tumor types (i.e., lymphosarcoma vs. lymphoma vs. synovial sarcoma) [43].

**Table 4:** Major Findings of Included Studies for Aim 2B: diagnostic performance of DWI with apparent diffusion coefficient (ADC) for grading of soft tissue tumors

Study (First Author et al., Year)	Imaging modalities, Sequence	Sensitivity	Specificity	Number of Tumors (N)	Tumor Type	Number of Readers	Inter-reader Analysis	Intra-reader Analysis	Minimum ADC (×10−3 mm2/s)	Mean/Median ADC (×10−	Maximum ADC (×10−3 mm2/s	Summary of Findings
Assy et al., 2022 [40]	MRI, DWI	NA	NA	52 total (24 low grade, 28 high grade)	Benign vs malignant	3	NA	NA	0.80 (highgrade STS)	Low-grade: 1.24 ± 0.414, High-grade: 0.921 ± 0.308	NA	ADC mean significantly lower in high-grade STS (p = 0.000). Tumor margins, necrosis, and peri-tumoral enhancement were predictors of high-grade STS.
Chhabra et al., 2019 [45]	MRI, DWI	NA	NA	51 total tumors	Malignant tumors	4	ICC: 0.60–0.98	NA	Grade III: 0.71	Grade I: 1.6 ± 0.59, Grade III: 0.9 ± 0.34	NA	Mean ADC is significantly lower in grade III than in grade I or II (p < 0.000). Longitudinal dimension larger in grade I than grade III (p < 0.000).
Manikis et al., 2022 [42]	MRI, DWI (Monoexponential, Biexponential, Stretchedexponential, DKI)	NA	NA	28 total tumors	Malignant tumors	NA	ICC = 0.916–0.966	NA	0.47	Low-grade: 1.08 ± 0.26, High-grade: 0.47 ± 0.32	NA	The skewness of metaDslow, overall-meta-D, and the IQR of meta-Dapp differentiate low- and highgrade sarcomas with AUC>89% (p < 0.05). Highgrade sarcomas showed lower ADC values (0.47 ± 0.32 × 10−3 mm²/s) compared to low-grade sarcomas (1.08 ± 0.26 × 10−3 mm²/s). The IQR represents heterogeneity of diffusion within the tumor; a higher IQR is often seen in more aggressive tumors.
Lee et al., 2019 [36]	DWI, DCEMRI	75	60	36 total tumors	Malignant soft tissue tumor	2	ICC =0.951	Not specified	0.67 × 10−3 mm²/s (high proliferation group)	Low proliferation: 1.20 × 10−3 mm²/s, High proliferation: 1.08 × 10−3 mm²/s	1.16	ADCmean inversely correlated with Ki-67 LI (ρ = −0.333, p = 0.047). A cutoff ADCmean of 1.16 × 10−3 mm²/s had 75% sensitivity and 60% specificity (AUC = 0.712, p = 0.014) for differentiating low- and high-proliferation tumor groups.
Hong et al., 2020 [44]	MRI + Texture analysis	94	67	42 total tumors	Malignant soft tissue tumors	1	NA	NA	NA	NA	NA	Texture features (Contrastenhanced=CE T1 mean, CE T1 skewness, CE T1 contrast) significantly differentiated between high- and low-grade sarcomas (AUC > 0.7, p < 0.05).
Gowda et al., 2023 [43]	MRI, DWI	NA	NA	78 total tumors	Malignant soft tissue tumors	2	ICC = 0.65–0.98	NA	0.2 (lymphoma) sarcoma)	1.5 (pleomorphic sarcoma), 1.1 (synovial sarcoma)	NA	Liposarcomas had higher mean ADC, while lymphomas and synovial sarcomas had lower values (p<0.05).

*ADC* apparent diffusion coefficient, *AUC* area under the curve, *CI* confidence interval, *DCE-MRI* dynamic contrast enhanced MRI, *DWI* diffusion-weighted imaging, *D *diffusion, *ICC* intraclass coefficient, *IQR* inter-quartile range, *IVIM* intravoxel incoherent motion, *PPV* positive predictive value, *NA* not available, *NPV* negative predictive value, STS- soft tissue sarcoma

Recent investigations into advanced MRI techniques have also demonstrated the ability for parametric meta-maps derived from multi-model DWI (including mono-exponential and kurtosis models) in differentiating STS grades, with specific histogram metrics achieving AUC exceeding 89% [32, 42]. ADC has also been inversely correlated with proliferation indices such as Ki-67, with lower ADC values being associated with higher Ki-67 levels in STS (*p* = −0.333, *p* = 0.047) [36]. Texture analysis may also be utilized to improve differentiation, with texture features such as T2 mean, T1 standard deviation, contrast-enhanced T1 skewness, and difference variance demonstrating significant differences, with AUCs greater than 0.7, indicating good discriminatory power. Additionally, qualitative features such as signal intensity on T1-weighted images and tumor margins on fat-suppressed contrast-enhanced T1-weighted images were significantly different between grades [35].

## Discussion

### Aim 1: Diagnostic accuracy of MRI in STS

MRI is the diagnostic modality of choice in differentiating benign and malignant soft tissue tumors, with both non-contrast and contrast-enhanced sequences having a significant role. Non-contrast T1W and T2W imaging help with identifying tissue characteristics and composition, which in turn help the radiologist differentiate between benign and malignant tumors. Contrast-enhanced sequences provide further information to help guide decision making when diagnosing soft tissue tumors. Previous studies have demonstrated different enhancing patterns when examining these tumors, with benign tumors typically having homogenous enhancement patterns and malignant tumors more likely to demonstrate inhomogeneous patterns [46]. Combining contrast enhanced imaging with nonenhanced static imaging can greatly improve diagnostic accuracy by providing key information on structure and metabolic activity as well as for post-operative monitoring of tumor recurrence [47, 48].

The findings of our review reveal significant variability in the sensitivity and specificity of MRI, reflecting the challenges inherent in the imaging evaluation of STS. As mentioned in our results section, sensitivity ranged from 61 to 100%, specificity ranged from 37 to 97%, PPV ranged from 32 to 94%, and NPV ranged from 87 to 100%. While sensitivity and NPV were on the higher end, there was marked variability on the specificity and PPV. This suggests that MRI alone may not be a reliable tool for diagnosing benign vs. malignant tumors and would be best supplemented with additional testing. Many studies that were included in our review have touched on this. MRI is the gold-standard in identifying these tumors and is an irreplaceable tool for assessment; however, results can be significantly improved by identifying specific parameters [16, 19], incorporating additional imaging such as ultrasound [17], and utilizing the ST-RADS classification system [20].

### Aim 2a: Sensitivity and specificity of DWI in differentiating between benign and malignant soft tissue tumors

As discussed previously, DWI and ADC values can play a role in differentiating between benign and malignant soft tissue tumors [49]. Malignant tumors typically have lower ADC values compared to benign tumors as evidenced when examining lipomatous tumors and PNSTs [33, 38]. It would be simple to conclude that any tumor with a low ADC value should automatically be concerning for malignancy; however, this is a bit more complicated as Einarsdóttir et al. noted substantial overlap in ADC values between benign and malignant lesions, suggesting that ADC alone may not always provide a clear distinction. An exception is round cell tumors such as lymphoma, where very low ADC values are observed [30, 43]. This is further complicated by the fact that there are no standardized ADC values across the studies that were included. It is difficult to standardize these given the variation between MRI scanner models, acquisition protocols, software, etc. utilized in different institutions.

Different areas of the body may also have different ADCs given changes in cellular composition [50, 51]. Soft tissue tumors occur in different parts of the body, which may impact results for the ADC values calculated. Alongside direct measurement, variations in post-processing methods (PPM) can impact the final value [52]. A study by Zeilinger et al. examined 65 tumors across the body and assessed four different PPM in final ADC values. They ultimately found that across these four PPMs, there was a significant mean coefficient variation of 7.2%. This study highlights the need for standardized ADC PPM for comparison of tumors even within the same institution [42]. Further work on this is necessary to help provide more reliable ADC values to aid with differentiating soft tissue tumors.

DWI can be improved by utilizing advanced imaging techniques. Wang et al.’s meta-analysis provided a comprehensive overview, highlighting that standard DWI using ADC values achieved moderate diagnostic performance, with pooled sensitivity and specificity of 0.80 and 0.63, respectively [32]. However, the inclusion of advanced DWI techniques such as IVIM and DKI improved diagnostic accuracy, particularly in non-myxoid tumors. This underscores the potential of advanced DWI methods to refine diagnostic criteria and reduce the rate of false positives.

Incorporating DWI can also improve outcomes for surgical patients. The study by Hong et al. highlighted the added value of DWI in assessing tumor margin infiltration in STS [35]. The incorporation of DWI into conventional MRI protocols significantly improved specificity for detecting infiltrative margins, which is critical for surgical planning and reducing the risk of local recurrence. Zampa (2022) also demonstrated the utility of DWI in post-surgical follow-up, where it provided higher diagnostic confidence in distinguishing between local recurrence and post-surgical changes [37].

### Aim 2b: Discriminatory power of ADC measurements in tumor grading

Tumor grading is an important task for the reading radiologist. As discussed previously in the results section, using DWI in the grading of STS, the findings consistently demonstrate that ADC values are inversely correlated with tumor grade, making them a valuable non-invasive biomarker for differentiating between low- and high-grade sarcomas. This differentiation is important since high-grade tumors often require more aggressive management strategies. Clinicians rely heavily on imaging reports to guide medical decision making; if diagnoses can be made with more confidence by the radiologist, patients can avoid unnecessary treatment/workup and receive expedited care.

Beyond traditional ADC metrics, the study by Manikis et al. introduced advanced DWI models and parametric meta-maps, which significantly improved the differentiation between low- and high-grade STS [42]. By incorporating models such as mono-exponential, bi-exponential, stretched-exponential, and diffusion kurtosis, the researchers were able to achieve area under the curve (AUC) values exceeding 89%. These advanced techniques provide a more detailed understanding of tumor microstructure, offering both visual and quantitative insights that enhance the accuracy of tumor grading.

In addition to ADC values, the study by Lee et al. explored the relationship between DWI and the Ki-67 labeling index, a marker of cellular proliferation [36]. The inverse correlation between ADC values and Ki-67 suggests that lower ADC values are indicative of higher proliferative activity, which is often seen in more aggressive tumors. This finding supports the broader application of DWI as a non-invasive imaging biomarker for tumor biology, with potential implications for predicting tumor behavior and response to therapy. If ADC values are standardized for specific tumors, ADC could potentially serve as a surveillance tool for tumors post-treatment without the overt need for other biomarkers. Future work may examine other biomarkers (p53, RB1, NF1, etc.) and their correlation with ADC values.

### Heterogeneity in the literature

As mentioned previously, we found significant heterogeneity across the studies included within our work. These differences may be further explained by variations in MRI protocols (e.g., field strengths, differing b-values in DWI, and processing protocols), inconsistent reporting of ADC cutoffs alongside lack of standardization, and differences in patient populations with regards to tumor locations, subtypes, and patient demographics. For example, different institutions utilize MRI machines with different field strengths, acquisition parameters, and interpretation criteria, which can lead to inconsistencies in reported sensitivity and specificity. This heterogeneity suggests there would be a low number of studies available for reliable pooling if a meta-analysis were to be considered, and greater identification and control for different factors would be needed. Further studies may look at individual tumor subtypes and control for patient characteristics to better allow for meta-analysis.

Future studies may also aim to assess the utility of other imaging modalities such as MRI and MR spectroscopy in differentiating tumor types. Such imaging modalities can provide valuable information as evidenced by numerous studies [44, 53]. CE-MRI provides critical information to distinguish tumor architecture such as solid components, necrotic areas, etc. MR spectroscopy can provide additional information on metabolic profiles to better assess tumor types.

### Applications

The findings in this review emphasize the growing need for judicious imaging in clinical practice. While MRI will remain the gold standard for soft tissue tumor assessment, identifying specific imaging characteristics that confidently classify lesions can help clinicians streamline the diagnostic process. Improved efficiency in this reduces the economic, physical, and time burden on patients by avoiding unnecessary imaging and invasive biopsies that carry their own risks (infection, bleeding, etc.).

Supplemental imaging modalities may be able to refine this process. For example, ultrasound can be a useful adjunct, particularly in low-resource areas as a screening tool prior to escalating to MRI. Additionally, PET/CT has emerged as a valuable supplemental tool for assessing metabolic activity and aiding in the differentiation of benign from malignant lesions [2]. Dynamic contrast-enhanced MRI (DCE-MRI) also holds potential added value by providing information on tumor vascularity and perfusion characteristics that may further help distinguish benign from malignant lesions and assist in treatment response assessment.

As with many other fields, the future of STS characterization will inevitably involve the integration of artificial intelligence (AI). One study by Hirozane et al. compared the efficacy of an AI model in differentiating tumors compared to an orthopedic oncologist and a radiologist [54]. They ultimately found the AI model to have comparable or higher accuracy (AUC 0.91) relative to the orthopedic oncologist (AUC 0.73) and radiologist (AUC 0.83) but had markedly greater speed (400 × faster than human) [54]. With further refinement, AI can be a powerful asset in differentiation by utilizing large datasets to improve diagnostic certainty. Overall, the future holds many exciting opportunities for improvements in our identification of benign and malignant soft tissue tumors to ultimately better serve our patients.

To conclude, this multi-aimed review focused on the diagnostic challenges associated with the MRI imaging of STS. The reviewed literature suggests that MRI offers high sensitivity and specificity in excluding malignancy but is hampered by a lower positive predictive value, highlighting the need for additional diagnostic methods. Furthermore, the integration of DWI into MRI protocols may enhance diagnostic accuracy, particularly by increasing specificity and confidence in distinguishing between benign and malignant tumors. Moreover, ADC values from diffusion-weighted imaging appear to inversely correlate to the tumor grade, suggesting utility in differentiating between low- and high-grade sarcomas. These findings highlight the important role of MRI and DWI in the diagnosis and grading of STS, while also underscoring the need for comprehensive approaches to reduce diagnostic uncertainties.

## Data Availability

It can be made available upon request and necessary ACR approvals.
